# Approved Societies and Hospital Treatment

**Published:** 1922-05

**Authors:** F. Squires


### APPROVED SOCIETIES AND HOSPITAL TREATMENT.

**By F. SQUIRES.**

UNDER the above scheme, over £250,000 per annum is available for distribution among the voluntary hospitals of the country, and the aim of any method of keeping the records should be to make the claim as complete as possible. The following method is proving satisfactory in practice at a large Midland hospital.

The present stage of the scheme involves only certain Approved Societies, but there is every reason to believe that additional Societies will eventually participate in the scheme. It is therefore desirable that full records be kept of all insured persons admitted to hospital for in-patient treatment, and the collection of the necessary information should begin in the Admittance Department. It will be found in practice that few patients, either on account of their condition on admittance or through not having the necessary documents in their possession, are unable to supply the particulars. The patient or his or her friends should be asked by the Admission Officer whether the patient pays State Insurance, and if the answer is in the affirmative, the officer will enter the word INSURED in the Admission Book and hand out a form requesting the following particulars: Name, Age, Address, Insurance Society, Section, Membership Number, and Name and Address of Insurance Agent. A list of patients insured is then abstracted from the Admission Book and entered into a special book. On completion, the form is returned to the hospital and ticked off in this register. By this means forms mislaid or not returned can easily be noted and steps taken to trace them. The forms are then filed under the different Societies and those of Societies included in the scheme are entered into a further book, the "Society" Book, ruled in the form required for rendering claims. A form is issued by all the large Societies and claims will only be accepted in respect of those patients for whom the hospital holds these forms.

It is advisable to write to the local offices of Societies to ascertain whether they wish applications for the necessary form to be made to them or direct to the agent concerned. It is a saving of time to write to them direct, but some Societies prefer the first alternative. The application should be in the form of a letter giving full particulars of the patient, including the date of admission, and requesting if the patient is entitled to "Additional Benefits," that the form required be sent to the institution. Upon the receipt of a form, the further particulars are entered into the "Society" Book. Ordinary exercise books ruled as required are quite suitable for the records.

**National Deposit Friendly Society's Scheme.**

The application is forwarded to the local agent, who supplies a perforated form (H.O.), the second part of which is returned to the Head Office, London, upon the patient's discharge, or at the end of each month's treatment. A grant is made at the rate of £1 1s. per week or part of a week.

**National Insurance Beneficent Society's Scheme.**

The first step is taken by the Society, but the Society should be informed of a member being admitted. A list of the Societies under this scheme will shortly be issued through the medium of the British Hospitals Association, and the grant is similar to that of the foregoing scheme.

**Prudential Approved Society's Scheme.**

This Society has four sections, for men, women, domestic servants, and agricultural and rural workers respectively. Separate claims and therefore separate sections in the "Society" Book are required. The ruling of these sections should be: Membership Number, Surname, Christian Name, Admission and Discharge dates, Weeks and Days patients are under treatment. The claim is made in duplicate four times a year, March 31, June 30, Sept. 30, and Dec. 31, patients remaining under treatment on those dates being entered to that date, and the particulars carried forward to the next period. The names should be placed in numerical order of membership. A grant is made for the total number of weeks of treatment, the odd days in the total (which cannot amount to more than six) being ignored. The admission and discharge days are counted as one day. The form required is Form A.1.

**National Amalgamated Society's Scheme.**

This amalgamation of Approved Societies includes the Albion Friendly, Bolton; Britannic; British Legal; Hearts of Oak; London and Manchester Industrial; Pearl; Pioneer Life; Refuge; and Royal London Mutual Societies. The "Society" Book is ruled similarly to that for the Prudential Society, with the following additional columns after that for Christian Name: District, Company. This information will be found on the form of authority (1589). Separate claims and sections in the book are required for men and women respectively. The time for rendering claims and the grant is similar to that of the Prudential Scheme. This scheme does not apply to members resident in Wales.

Under the last two schemes, over £200,000 is available annually for distribution.

**Western Mutual Assurance Society's Scheme.**

No form of authority is required to support the claim. A list of members treated as in-patients, giving full particulars of membership number, &c., is forwarded half-yearly and a grant is made on the total number of in-patient days.

The nominal date on which all these schemes came into force is January 1, 1922, and all patients in the hospital on that date should be included in the claims. The scheme of the National Deposit Society has been in operation since July 4, 1921.

**Royal Liver Friendly Society.**

This Society has now adopted a scheme similar to those of the large industrial societies, and the procedure for claiming grants will be exactly similar to that indicated in connection with the Prudential.

**Liverpool Victoria Friendly Approved Society.**

From July, 1921, for a period of five years, the Society has agreed to pay 3s. per day for members in hospital. A list of members treated, giving full insurance particulars, is to be forwarded each month to the Society. Those hospitals which have the necessary records of insured patients treated in 1921 can, of course, claim from the commencing date of this scheme.

It will be found that many patients are averse from giving the particulars required, mainly through ignorance of the scheme. It is therefore advisable to make known through the Press and the Hospital Saturday Committees that the giving of this information in no way affects any benefit to which patients may be entitled under the Health Insurance Act.

The British Hospitals Association suggests that a list of members treated in the wards should be forwarded to all approved societies, in the hope that those societies which have not adopted a scheme to benefit the voluntary hospitals may realise the enormous amount of work done for insured persons. As most societies have by now decided upon some method of disposing of their surplus funds, no large success may be expected from such action.

---

At the annual meeting of the Hospital Saturday Fund the income fro 1921 was stated at £107,147, the largest amount collected in any one year.

We have received for the editorial table a useful accessory in the form of an ash-tray and paper-weight in heavy glass, which the Relay Automatic Telephone Company, Ltd., of Marconi House, Strand, W.C.2, are presenting to their clients.

For many years the "Crystal Palace" marking ink of Messrs. John Bond (London), Ltd., has been esteemed in the linen rooms of hospitals and other institutions. Users of the excellent original preparation will be glad to know that the new "Non-heat" variety is equally effective, and saves time and trouble by requiring no ironing. A handy linen stretcher and a penholder with several nibs are presented with the shilling and larger sizes, which can be obtained from all stationers, chemists and stores.

The American Occupational Therapy Association now has its own official organ, "Archives of Occupational Therapy," for the publication of articles hitherto scattered in many periodicals. It is hoped that other countries as well as the United States will be represented in its pages. The journal is published bi-monthly by the Williams & Wilkins Company, Baltimore, U.S.A. The first (February) number contains an article on "The Philosophy of Occupation-Therapy," by Professor Adolf Meyer of Johns Hopkins University, and interesting papers on recreational and other activities for mental patients, tuberculous and cardiac subjects, for children in hospital, and for the bed-ridden or house-bound.

